# Abortion laws reform may reduce maternal mortality: an ecological study in 162 countries

**DOI:** 10.1186/s12905-018-0705-y

**Published:** 2019-01-05

**Authors:** Su Mon Latt, Allison Milner, Anne Kavanagh

**Affiliations:** 1 Melbourne School of Population and Global Health, The University of Melbourne, Melbourne, Australia; 2 Centre for Health Equity, Melbourne School of Population and Global Health, University of Melbourne, Melbourne, Australia

**Keywords:** Maternal mortality, Abortion laws, Ecological study, Panel data, Policy

## Abstract

**Background:**

Unsafe abortion is one of the commonest causes of maternal mortality. Abortion-related maternal deaths are higher in countries with the most restrictive abortion laws. We assess whether maternal mortality varies within and between countries over time according to the flexibility of abortion laws (the number of reasons a woman can have an abortion).

**Method:**

We conducted an ecological study to assess the association between abortion laws and maternal mortality in 162 countries between 1985 and 2013. Aggregate-level data on abortion laws and maternal mortality were extracted from United Nations (UN), and World Health Organization’s (WHO) database respectively. A flexibility score of abortion laws (Score 0–7) was calculated by summing the number of reasons for which abortion was legally allowed in each country. The outcome was maternal mortality ratio (MMR), which represented maternal deaths per 100,000 live births. MMR was modelled as a continuous variable and flexibility score as an ordinal ranked variable (categories 0–7 with 0 as the reference, and < 3 vs > = 3). We used fixed effects linear regression models to estimate the association between flexibility score and MMR, adjusting for gross domestic product per capita (GDP per capita), and time in five-year intervals.

**Results:**

Compared to when a country’s flexibility score was < 3, maternal deaths were reduced by 45 per 100,000 live births (95% CI: -64, − 26) when the flexibility score increased ≥3, after adjusting for the GDP per capita and five-year time intervals. With the exception of a flexibility score 6, MMR was lower when higher than zero. This may indicate the role of other country- specific effects.

**Conclusion:**

This study provides evidence that abortion law reform in countries with restricted abortion laws may reduce maternal mortality.

**Electronic supplementary material:**

The online version of this article (10.1186/s12905-018-0705-y) contains supplementary material, which is available to authorized users.

## Background

Maternal mortality remains a global challenge, despite efforts to achieve Sustainable Development Goals (SDGs) three of reducing maternal mortality. There were over 300,000 maternal deaths in 2015 among WHO member countries, corresponding to an overall maternal mortality ratio (MMR) of 216 (Uncertainty Interval (UI), 207 to 249) per 100,000; 99% of maternal deaths occur in developing regions [1]. Nearly 8 % of maternal deaths worldwide are abortion-related and 99.5% of these occurring in developing regions [1, 2].There is strong evidence linking unsafe abortions with increased maternal morbidity and mortality [3–7] and most abortion-related maternal deaths are due to unsafe and illegal abortions [8]. Although the overall abortion rate has declined, the proportion of unsafe abortions is increasing, especially in developing regions [3]. Unsafe abortions are most common in countries with restrictive abortion laws [9]. This suggests that improving abortion law reform could reduce maternal mortality, however, we do not have a rigorous evidence-base on which to support this premise.

Throughout the world, there is considerable variation in abortion laws. In countries such as Sierra Leone, Afghanistan, Nigeria, Lao, and Myanmar, abortion is only legally allowed if a woman is in life-threatening situations. The policies are least restrictive in developed countries such as Australia, France, Germany, Italy, Canada, and United States, where a woman can request legal abortion for a range of reasons including physical or mental health, rape, fetal impairments or economic conditions [10]. The disparity in abortion-related maternal mortality could partly be explained by differences in abortion laws with women in countries with more restrictive laws being less able (or unable) to access to reproductive health care and safe abortion services.

There is little or no research about abortion laws and their impact on maternal mortality. Most studies have assessed change in trends of maternal mortality within countries after legalization without accounting for the other factors, such as levels of female education, which also change with time. For example, Handerson et.al [11] conducted a retrospective medical chart review in Nepal after abortion law was reformed to legally permit abortion for rape or incest, physical or mental health reasons. They found that the reformation of abortion laws in Nepal appeared to be associated with a significant decline in the rate of abortion-related morbidities, such as serious infections and systematic complication. This study did not address possible impacts on maternal mortality [11]. Of those studies that have been conducted, most have found a significant decrease in maternal mortality when abortion laws became less restrictive [11–16]. However, one study conducted in Chile found that maternal mortality was decreased after abortion became completely illegal in Chile [13]. Only one study was conducted in more than one country, where changes in abortion laws and maternal mortality were assessed in Romania, South Africa, and Bangladesh [12]. This study found a downward trend in maternal mortality after the liberalization of abortion laws [12].

While existing studies provide weak evidence of an association between more flexible abortion laws and reduced maternal mortality, all but one have been conducted within one country and none of the studies take account of confounding variables that change over time such as economic conditions and female education. In order to fill this gap in research, we conducted an ecological analysis using data from 162 countries on abortion laws and maternal mortality between 1985 and 2013 to estimate the association between the flexibility of abortion laws and maternal mortality at a country level.

## Methods

All data were at an aggregate level by country and year. The study population was selected from the WHO and UN member countries with available data. The data on abortion laws were obtained from the United Nations (UN) Population Division Database [17] and maternal mortality data were obtained from the WHO Global Health Observatory Database [18]. Maternal mortality data was available for 183 countries between 1985 and 2013. There were175 countries with data on abortion laws over the same time period. The final sample included 162 countries with information on both abortion laws and maternal mortality between 1985 and 2013. Data extraction was done by S.M in May 2016.

### Definition of variables

Information on the source, definition, and completeness of data for the exposure, outcome and covariates are available in Additional file 1: Table S1.

#### Exposure variable

The exposure of interest was the flexibility of abortion laws in each country for each year. The United Nations Population Division database, which recorded abortion laws around the world from 1963 to 2013, [19] had yearly data on abortion law policies between 1985 and 2005 and two-yearly thereafter (2007, 2009, 2011, and 2013). These data were manually extracted on 10th March 2016 using the “manual data query” function from “World Population Policies” database [17]. We classified the abortion laws according to the abortion law categories of UN population policy database [17, 19]. Grounds for which abortion is legally allowed in the country were coded in the database as:

1. Intervention to save the life of the woman (life grounds)
2. Preservation of the physical health of the woman (narrow health grounds)
3. Preservation of the mental health of the woman (broad health grounds)
4. Termination of pregnancy resulting from rape or incest (juridical grounds)
5. Suspicion of fetal impairment (fetal defect)
6. Termination of pregnancy for economic or social reasons (social grounds)
7. Availability upon request: abortion permitted on all grounds

We summed the number of grounds/reasons for which abortion was legally allowed in each country from 1985 to 2013 to give a flexibility score ranging from zero to seven. A flexibility score of zero means abortion is not legally allowed for any reason and a flexibility score of seven means abortion is legally allowed for any of the aforementioned grounds.

#### Outcome variable

The outcome was maternal mortality ratio (MMR), which is the number of maternal deaths per 100,000 live births [1] (Refer to Additional files 1, 2, 3 and 4). Maternal mortality ratios in reproductive-aged (15–49 years) women were accessed from the World Health Organization Global Health Observatory database [18]. MMR was analyzed as a continuous outcome variable. We used MMR rather than abortion-specific maternal mortality, as abortion is a highly sensitive issue, especially in countries with restrictive abortion laws, where abortion is only allowed for life-threatening conditions. As the medical professionals and women performed the induced abortions can be imprisoned in places where this is illegal, we anticipated underreporting for induced abortions and abortion-related maternal mortality in such countries. This was supported by a recent systematic review of the measurements of abortion-related maternal mortality, where the results indicated underestimation and misclassification of abortion-related maternal mortality due to poor quality measurement [20].

#### Other covariates

Data on potential confounders were also extracted from the United Nation Population Division database [17] and the World Bank database [21–24]. Covariates included: female primary completion rate (%); female literacy rate (%), health expenditure per capita (in US dollars), and Gross Domestic Product per Capita (GDP per capita in US dollars). Details of data sources and definitions can be found in Table 1.

**Table 1:** Data sources and Definitions

Variable	Source	Definition	Time period
Female primary completion rate (Female %)	The World Bank Education database [21]	Total number of new female entrants in the last grade of primary education, regardless of age, expressed as a percentage of the total female population of the theoretical entrance age to the last grade of primary. This indicator is also known as “gross intake rate to the last grade of primary education.” The ratio can exceed 100% due to over-aged and under-aged children who enter primary school late/early and/or repeat grades.	1985–2013 (50% complete)
Female Literacy Rate (Female, %)	The World Bank Education database [22]	Percentage of females age 15 and above who can, with understanding, read and write a short, simple statement on their everyday life. This indicator is calculated by dividing the number of female literates aged 15 years and over by the corresponding age group population and multiplying the result by 100.	1985–2013 (only 10% complete)
Health Expenditure per capita (current US$)	The World Bank health database [23]	Total health expenditure is the sum of public and private health expenditures as a ratio of total population. It covers the provision of health services (preventive and curative), family planning activities, nutrition activities, and emergency aid designated for health but does not include provision of water and sanitation. Data are in current U.S. dollars.	1995–2013 (60% complete)
Abortion law indicators	United Nations Population Division Database, Department of Economic and Social Affairs [17]	Refer to the exposure variable.	1985–2013 (100% complete)
Maternal mortality ratio	World Health Organization (Global Health Observatory Database) [18]	Maternal mortality ratio (MMR) Maternal mortality ratio is defined as maternal deaths per 100,000 live births.	1985–2013 (100% complete)
GDP per capita	World bank, World Development Indicator Database [24]	GDP per capita is gross domestic product divided by midyear population. GDP is the sum of gross value added by all resident producers in the economy plus any product taxes and minus any subsidies not included in the value of the products. It is calculated without making deductions for depreciation of fabricated assets or for depletion and degradation of natural resources. Data are in current U.S. dollars.	1985–2013 (93% complete)

#### Missing data

Table 1 shows that there were high proportions of missing data for female primary education, female literacy rate and health expenditure per capita. There was high co-linearity between female literacy rate and female primary completion rate, and between GDP per capita and health expenditure per capita. Therefore, we included the variables with the fewer missing values (Refer to Table 1) in the regression models described below (i.e. GDP per capita and female primary completion rate).

### Statistical analysis

First, we present the summary statistic by five- year time intervals for MMR, flexibility score and GDP per capita. The dataset was treated as a “panel dataset” [25] because the variables were recorded for the 162 countries at different points of time (i.e. 1985 to 2013). We conducted fixed effects linear regression modelling to assess the association between flexibility score of abortion laws and MMR within country before and after adjustment for economic income level (GDP per capita) and female education (female primary completion). Models were fitted separately with or without female education because of the high proportion of missing data on this variable (50%). We modelled the flexibility score as a seven-level categorical variable with zero as the baseline and also as a binary variable < 3 vs 3 or more based on the distribution of the variable [26]. We also adjusted for time in five-year intervals to account for secular trends in maternal mortality and the flexibility score over time.

Fixed effects regression models have been widely used in ecological studies to analyze time series cross-sectional (panel) data [27, 28]. Fixed effects models assess within-country differences (i.e. each country is its own control). For example, the difference in MMR within country when the flexibility score was zero compared to when greater zero. This approach removes confounding from variables that do not change over time [26] For example, ethnic and religious composition of a country may be relatively stable over time. The “Hausman Test” showed that fixed effects rather than random effects regression was required because there was evidence of significant unmeasured time-invariant confounding between countries [29]. All analyses of fixed effects regression models were undertaken in STATA version 13.0 using “xtreg,fe”.

## Results

The average MMR, average flexibility score, and average GDP per capita for each country are presented in Additional file 1: Table S1. Table 2 presents summary statistics for MMR, flexibility score and GDP in five-year intervals. MMR decreased over time and mean flexibility score increased slightly. In all time intervals, median estimates were less than mean MMR estimates, showing the positively skewed distribution of MMR.

**Table 2:** Summary measures of MMR, flexibility scores and GDP per capita in 162 countries by five-year time intervals

		Time Interval					
		1985–1989	1990–1994	1995–1999	2000–2004	2005–2009	2010–2013
MMR (Maternal deaths per 100,000 live births)	Mean	367	328	296	259	219	186
	Median	124	97	88	74	67	59
	SD	467	446	413	359	298	253
	Minimum	5	4	4	3	3	3
	Maximum	2558	2902	2895	2649	1986	1580
Flexibility Score (0–7)	Mean	3.67	3.82	3.86	3.84	3.90	4.10
	Median	3	3	3	3	4	4
	SD	2.50	2.47	2.47	2.50	2.50	2.50
	Minimum	0	0	0	0	0	0
	Maximum	7	7	7	7	7	7
GDP per Capita (current US$)	Mean	4220	5386	6442	7353	11,310	14,367
	Median	1179	1312	1633	1967	3467	5470
	SD	6269	8667	10,119	11,625	17,250	20,958
	Minimum	97	65	65	106	141	240
	Maximum	31,664	45,935	53,358	74,970	104,841	113,726
Female primary completion rate (Female, %)	Mean	66	70	76	81	85	89
	Median	71	81	88	94	95	97
	SD	32	32	29	29	23	20
	Minimum	4	6	7	13	20	26
	Maximum	135	120	128	188	134	116

In 1985, four countries had a flexibility score of zero (El Salvador, Guyana, Indonesia, and Malta), and in 2013, five countries had a flexibility score of zero (Chile, Dominican Republic, El Salvador, Malta, and Nicaragua). In 1985, 43 countries had a flexibility score of seven and this increased to 52 countries in 2013. Between 1985 and 2013, Finland had the lowest MMR (approximately five per 100,000 live births in 1985 and three per 100,000 live births in 2013). Sierra Leone had the highest MMR (approximately 2352 per 100,000 live births in 1985 and 1460 per 100,000 live births in 2013).

The specific reasons allowed for legal abortions in each flexibility score (Score 0–7) were also assessed (See Additional file 1: Table S2). For example, for a flexibility score of two, the combination of reasons was: life-threatening+ rape (47%), or life-threatening+ physical health (35%), and life-threatening+ fetal impairment (17%).

### Fixed effects regression models

The univariate fixed effects regression models indicated that higher flexibility scores of abortion laws were associated with lower maternal mortality ratios (MMR). When flexibility score was fitted as a categorical variable (0–7) there was evidence of a reduction in maternal mortality of between 85 and 144 per 100,000 live births for flexibility scores of three or more compared to zero. When flexibility score was fitted as a binary variable, there were 109 fewer maternal deaths per 100,000 live births (95% CI -133, − 84) when the flexibility score was three or more compared to less than three.

After adjusting for GDP per capita and time intervals, the effects were slightly attenuated. However, the strong evidence that higher flexibility score of abortion laws was associated with lower MMR for all flexibility scores three or more compared to zero with the exception of flexibility score six. The estimated mean MMR was reduced in higher flexibility score group (> = score 3) compared to low flexibility score group (< score 3) by 45 after adjusting for GDP per capita and time intervals. (95% CI: -26, − 64).

Including female education in the models attenuated the estimates, although the same general trend was evident. After adjusting for GDP per capita, time intervals, and female education, when the flexibility score of abortion laws was three or higher, the mean MMR was 25 maternal deaths per 100,000 births lower (95% CI: -4, − 46) than when the flexibility score was lower than three. However, because there was a large amount of missing data on female education, the number of observations was much lower. The results of the univariate and multivariate fixed effects models are summarized in Table 3 and Table 4 respectively.

**Table 3:** Univariate fixed effects regression of the association between flexibility score and maternal mortality ratio in 162 countries, 1985–2013 (*N* = 4212, *n* = 162)

Flexibility score as a 7 level categorical variable (Baseline = Score 0)			
Score	Coefficient	95% Confidence interval	*p*-value
Score 1	−28	(−80, 24)	0.294
Score 2	−42	(− 106, 22)	0.198
Score 3	−144	(− 206, 83)	< 0.001
Score 4	−85	(− 149, −22)	0.009
Score 5	− 180	(− 240, − 120)	< 0.001
Score 6	−97	(− 186, − 8)	0.033
Score 7	− 138	(− 197, −78)	< 0.001
Constant	388	(336, 440)	< 0.001
Flexibility score as a binary variable (Score < 3 versus > = 3)			
Flexibility Score > =3	−109	(− 133, −84)	< 0.001
Constant	359	(343, 376)	< 0.001

Score1: abortion is legally allowed for only 1 reason, Score 2: abortion is legally allowed for 2 reasons, and so on. N = total no. of observations, n = number of countries

**Table 4:** The association between flexibility of abortion laws and maternal mortality ratio after adjusting for GDP per capita and time trends, 1985–2013

	Model adjusted for GDP and Time intervals (*N* = 3926, *n* = 161)			Model adjusted for GDP, FPC, and Time interval (*N* = 1990, *n* = 144)		
Flexibility score as a 7 level categorical variable (Baseline = Score 0)						
Score	Coefficient	95% Confidence interval	*p*-value	Coefficient	95% Confidence interval	*p*-value
Score 1	− 54	(−94, −14)	0.008	−13	(− 73, 48)	0.681
Score 2	−69	(−119, −20)	0.006	−26	(−95, 43)	0.454
Score 3	− 132	(−179, −85)	< 0.001	−50	(− 116, 16)	0.134
Score 4	−70	(−119, −21)	0.005	−11	(−75, 54)	0.748
Score 5	−119	(− 166, −73)	< 0.001	−65	(− 129, −2)	0.044
Score 6	−17	(−86, 51)	0.622	2	(−83, 87)	0.965
Score 7	−72	(− 118, −26)	< 0.001	−18	(−79, 43)	0.56
GDP	0.005	(0.004, 0.005)	< 0.001	0.004	(0.003, 0.004)	< 0.001
FPC				−1.8	(−2.1, −1.4)	< 0.001
1990–1994*	−42	(−51, −33)	< 0.001	−39	(−50, −27)	< 0.001
1995–1999	− 77	(−86, −68)	< 0.001	−72	(−84, −60)	< 0.001
2000–2004	−116	(− 126, − 107)	< 0.001	−96	(− 108, −85)	< 0.001
2005–2009	− 173	(− 184, − 163)	< 0.001	−134	(− 147, −120)	< 0.001
2010–2013	−220	(− 233, −206)	< 0.001	− 164	(− 182, −147)	< 0.001
Constant	419	(379, 459)	< 0.001	492	(429, 556)	< 0.001
Flexibility score as a binary variable (Score < 3 versus > = 3)						
Flexibility Score > = 3	−45	(−64, – 26)	< 0.001	−25	(−46, − 4)	0.022
GDP	0.005	(0.004, 0.005)	< 0.001	0.004	(0.003, 0.005)	< 0.001
FPC				−2	(−2, −1)	< 0.001
1990–1994*	−42	(−51, −33)	< 0.001	−39	(−50, −27)	< 0.001
1995–1999	−77	(−86, −68)	< 0.001	−73	(−84, −61)	< 0.001
2000–2004	−116	(−126, − 107)	< 0.001	−97	(− 109, −85)	< 0.001
2005–2009	−173	(− 184, −163)	< 0.001	− 135	(− 148, − 121)	< 0.001
2010–2013	− 219	(− 232, − 205)	< 0.001	− 165	(− 182, − 148)	< 0.001
Constant	369	(356, 383)	< 0.001	485	(456, 515)	< 0.001

*GDP* Gross Domestic Product Per Capita (US$), *FPC* Female Primary Completion (Female, %)

N = total number of observations, n = number of countries, *Baseline for five-year interval=1985-1989

## Discussion

In this large ecological study of abortion law and maternal mortality, we found that MMR decreased as a country increased its flexibility score. With the exception of a flexibility score of six, all countries that permitted abortion for at least one reason had lower MMR than when the flexibility score was zero. As we discuss below, this result is likely to reflect a low number of observations with this score.

When the flexibility score was modelled as a binary variable, we found that countries with a flexibility score of three or more, there were 45 less maternal deaths per 100,000 live births than when the flexibility score was less than three, after accounting for the GDP per capita and secular downward trends of maternal mortality.

Our findings that the adoption of flexible abortion laws was associated with a decrease in the countries’ maternal mortality is different from one study conducted in Chile between 1957 to 2007 [13]. The Chilean study found that maternal mortality decreased after abortion became completely illegal however they adjusted for variables such as sanitation and clean water that are unlikely to be confounders. They also adjusted for the total fertility rate which is likely to be a mediator rather than a confounder [13]. Our results are consistent with previous, smaller studies conducted in Nepal, United States, Romania, South Africa, Bangladesh, and Mexico [11, 12, 15].

There are a number of explanations for the association between the flexibility of abortion laws and maternal mortality. First, when abortion is legal and accessible within the health system, the quality of abortion services is improved, and thus reducing the incidence of unsafe abortions [14]. Second, it is possible that change in total fertility rates (TFR) may play a role in influencing maternity mortality. Although the exact mechanism of the association between TFR and maternal mortality is still unknown, countries with higher TFR have higher maternal mortality [30]. In a national household survey in Romania, TFR reduced after the restrictive abortion law was abolished in 1989 [31]. This is supported by a recent study in Mexico, which examined the effect of elective abortion program in Mexico city in 2007 [32]. Third, some effects can be mediated through the changes in the health-seeking behaviors of women. Women with an unwanted pregnancy will seek safe abortion services if they can request abortions legally.

Our findings suggest that the liberalization of abortion laws will reduce maternal mortality. In our sample of 162 countries, 48 countries had flexibility score less than three in 2013; it is possible that maternal mortality would reduce in these countries if legal, safe abortion was more readily available. However, it is important to acknowledge that it may take years for abortion law reform to impact on maternal mortality. In fact, the change of the abortion laws itself may not be sufficient to reduce maternal mortality. Abortion law reform must also be accompanied with improved access to safe abortion services, as well as improvement in community attitudes (e.g., reduction in stigma) towards these services.

The experience of abortion law reforms in Cambodia, Colombia, Ethiopia, Mexico City, Nepal and South Africa identified some pragmatic issues in transitioning from advocacy and passage of a law to implementation. Successful implementation of abortion laws reform requires public awareness of changes in the law, clinical and administrative guidelines and dissemination to standardize delivery of medical care, and creation and uptake of safe abortion services [33]. Additionally, women need to be aware of the change in the laws and have knowledge of how to access the safe abortion services. A recent systematic review found that women may be unaware, or have incorrect knowledge, about abortion laws in their countries even in countries with more liberal abortion laws. [34] Therefore, it is important to develop interventions to promote accurate information after legal access to abortion has been increased.

This study has some limitations which have to be discussed. First, it is possible that abortion law does not reflect access to safe abortion. For example, in Bangladesh in 2013, the flexibility score was one because abortion was only legally allowed if the pregnancy was life- threatening for the woman. However, ‘menstrual regulation’ services are widely available to women in Bangladesh. ‘Menstrual regulation’ involves vacuum aspiration within 6 to 10 weeks of a missed menstrual period without a pregnancy test [35]. Vacuum aspiration is one of the procedures used in abortion [36]. This means that women can request abortion services legally using a different name for the service. There is also the worldwide trend change from surgical abortions towards medical abortions by misoprostol since the 1990s. The study design addresses these limitations by adjusting time-periods in the analysis. In the Latin-America and Caribbean countries, the decline in abortion-related maternal mortality is observed due to access to safer abortion by using misoprostol as a medical abortion despite the most-restrictive abortion laws [37]. Despite highly restrictive abortion laws, access to the medical abortion increases access to safer abortion. Therefore, we also conducted a sensitivity analysis for the fixed-effects regression models adjusted for time and GDP by excluding 24 Latin-America and Caribbean countries. The sensitivity analysis showed a stronger association between the flexibility of abortion laws and the reduction of MMR (Table 5).

**Table 5:** The results of sensitivity analysis (*N* = 3309, *n* = 137)

Flexibility score as a 7 level categorical variable (Baseline = Score 0)			
Score	Coefficient	95% Confidence interval	*p*-value
Score 1	− 141	(−220, −61)	0.001
Score 2	−149	(−235, −63)	0.001
Score 3	− 253	(− 338, − 168)	< 0.001
Score 4	− 172	(− 261, −83)	< 0.001
Score 5	−232	(− 317, −146)	< 0.001
Score 6	−146	(− 254, −38)	0.008
Score 7	−197	(− 283, − 111)	< 0.001
GDP	0.005	(0.004, 0.005)	< 0.001
1990–1994	−47	(−57, −36)	< 0.001
1995–1999	−86	(−96, −75)	< 0.001
2000–2004	− 130	(−141, −120)	< 0.001
2005–2009	−196	(− 208, −184)	< 0.001
2010–2013	−249	(− 264, − 233)	< 0.001
Constant	564	(484, 643)	< 0.001
Flexibility score as a binary variable (Score < 3 versus > = 3)			
Flexibility Score > = 3	−82	(−106, −57)	< 0.001
GDP	0.005	(0.005, 0.006)	< 0.001
1990–1994	−47	(−58, −37)	< 0.001
1995–1999	−87	(−98, −76)	< 0.001
2000–2004	−132	(− 143, −121)	< 0.001
2005–2009	−198	(− 210, − 185)	< 0.001
2010–2013	− 248	(− 263, −233)	< 0.001
Constant	432	(415, 449)	< 0.001

*GDP* Gross Domestic Product Per Capita (US$)

N = total no. of observations, n = number of countries

Adjusted for Time and GDP

The flexibility score of six was not associated with lower mortality. This may be explained by having a fewer number of countries in the flexibility score of six. For instance, there were only five countries with score six in 1985 (Barbados, Finland, Greece, India, and Luxembourg) where there were only seven countries with score six in 2013 (Barbados, Finland, India, Fiji, Iceland, Luxembourg, and St. Vincent and the Grenadines). So, there is a possibility that the regression models did not have enough power to detect the difference between the two groups due to a relatively fewer number of countries in score six. Additionally, we did not have any information on some of the key variables. For example, there was no reliable data on gender equity which is likely to be a confounder. There was attenuation of the estimates when female primary completion rate was included in the models however 50% of data was missing for this variable (i.e. 50%).

This study has a number of important strengths. As far as we are aware this is the most comprehensive study of abortion law and maternal mortality ever conducted. We had a large sample of 162 countries over a 28-year time period, which included high, medium and low- income countries. We used an ecological study design – the most appropriate study design when ecological effects are of interest. An ecological study is particularly relevant when the level of inference is ecologic rather than individual, and when evaluating the effects of social processes or population interventions such as new programs, policies, or legislation [38].

Our use of fixed effects regression models is a significant advance. Fixed effects regression models allowed us to control for the possible confounding associated with country effects and reduce omitted variable bias [26] due to time-invariant variables. In contrast to previous studies [12, 15], we attempted to adjust for time-varying confounding by including GDP per capita and five-year time periods.

## Conclusion

In conclusion, our study demonstrates that maternal mortality is lower when abortion laws are less restrictive. Our results suggest that there is a need to reform abortion laws in the countries with the most restrictive abortion laws, and to provide safe abortion services to protect women from unsafe and illegal abortions. To improve our understanding of the associations between abortion law and maternal mortality and other women’s health issues we recommend that key country-level variables such as contraceptive prevalence, female education and gender equity are systematically recorded.

## Additional files

Additional file 1: Table summarizing the specific reasons for legal abortion for each flexibility score. This table describes a summary for the specific reasons allowed for legal abortions in each flexibility score (0–7). (DOCX 50 kb)

Additional file 2: Glossary and Definitions. Glossary and Definitions for the Uncertainty Interval, maternal mortality ratio (MMR), Maternal Death, and Live Birth. (DOCX 33 kb)

Additional file 3: Maternal Mortality Ratios (Mean) and the flexibility score of abortion laws (Mean score) in the sample countries, 1985–2013. Description of Data: The table summarizes the average score for abortion laws and average maternal mortality ratios for each sample country from 1985 to 2013. (DOCX 37 kb)

Additional file 4: List of Latin-American and Caribbean Countries excluded for Sensitivity Analysis. The list provides the name of 24 Latin-America and Caribbean countries which has been excluded to conduct sensitivity analysis. (DOCX 35 kb)

## Abbreviations

GDP per capita: Gross domestic product per capita
MMR: Maternal mortality ratio
SDG: Sustainable development goals
TFR: Total fertility rate
UI: Uncerntainty interval
UN: United Nations
WHO: World Health Organization
