# Supplementary material for: Abortion laws reform may reduce maternal mortality: an ecological study in 162 countries
Source: BMC Womens Health. 2019 Jan 5;19:1. doi: 10.1186/s12905-018-0705-y (PMC6321671; doi:10.1186/s12905-018-0705-y)
Supplement: Supplementary file 1 — Table summarizing the specific reasons for legal abortion for each flexibility score. This table describes a summary for the specific reasons allowed for legal abortions in each flexibility score (0–7). (DOCX 50 kb) [file 12905_2018_705_MOESM1_ESM.docx]

**Table showing Maternal Mortality Ratios (Mean) and the flexibility scores of abortion laws (Mean score) in the sample countries, 1985- 2013**

| Country | MMR  (Mean) | Flexibility Score  (Mean) | GDP per capita  (Current US$) |
| --- | --- | --- | --- |
| Afghanistan | 1114 | 1 | 341 |
| Albania | 51 | 5 | 1567 |
| Algeria | 190 | 3 | 2500 |
| Angola | 961 | 1 | 1401 |
| Argentina | 65 | 3 | 6533 |
| Armenia | 46 | 7 | 1244 |
| Australia | 8 | 7 | 25549 |
| Austria | 6 | 7 | 28913 |
| Azerbaijan | 57 | 7 | 1831 |
| The Bahamas | 60 | 3 | 16994 |
| Bahrain | 22 | 7 | 12820 |
| Bangladesh | 442 | 2 | 412 |
| Barbados | 46 | 6 | 10542 |
| Belarus | 25 | 7 | 2582 |
| Belgium | 8 | 6 | 27341 |
| Belize | 51 | 5 | 3063 |
| Benin | 555 | 2 | 452 |
| Bosnia and Herze | 22 | 7 | 2290 |
| Botswana | 261 | 4 | 3514 |
| Brazil | 84 | 2 | 4541 |
| Brunei | 33 | 1 | 19271 |
| Bulgaria | 20 | 7 | 2897 |
| Burkina Faso | 597 | 3 | 332 |
| Burundi | 1046 | 3 | 172 |
| Cambodia | 669 | 7 | 446 |
| Cameroon | 725 | 4 | 883 |
| Canada | 8 | 7 | 26567 |
| Central African | 1183 | 1 | 357 |
| Chad | 1334 | 1 | 375 |
| Chile | 41 | 0 | 5565 |
| China | 70 | 7 | 1379 |
| Colombia | 103 | 2 | 2695 |
| Comoros | 535 | 3 | 535 |
| Congo, Rep. | 607 | 1 | 1316 |
| Costa Rica | 39 | 3 | 4008 |
| Cote d'Ivoire | 726 | 1 | 879 |
| Croatia | 11 | 7 | 8554 |
| Cuba | 50 | 7 | 3214 |
| Cyprus | 13 | 5 | 16242 |
| Czech Republic | 9 | 7 | 9503 |
| Denmark | 10 | 7 | 35632 |
| Djibouti | 431 | 1 | 878 |
| Dominican Republic | 150 | 1 | 2534 |
| Ecuador | 132 | 4 | 2424 |
| Egypt, Arab Rep. | 78 | 1 | 1239 |
| El Salvador | 112 | 0 | 2013 |
| Equatorial Guine | 885 | 3 | 4927 |
| Eritrea | 1068 | 3 | 244 |
| Estonia | 31 | 7 | 8686 |
| Ethiopia | 975 | 3 | 205 |
| Fiji | 50 | 4 | 2593 |
| Finland | 5 | 6 | 29086 |
| France | 13 | 7 | 26575 |
| Gabon | 399 | 1 | 5537 |
| Gambia, The | 927 | 3 | 527 |
| Georgia | 37 | 7 | 1388 |
| Germany | 9 | 7 | 27932 |
| Ghana | 512 | 5 | 561 |
| Greece | 4 | 7 | 14792 |
| Grenada | 33 | 3 | 4138 |
| Guatemala | 171 | 1 | 1634 |
| Guinea | 961 | 3 | 411 |
| Guinea-Bissau | 798 | 1 | 287 |
| Guyana | 200 | 1 | 1186 |
| Haiti | 546 | 4 | 460 |
| Honduras | 197 | 1 | 1106 |
| Hungary | 19 | 7 | 7513 |
| Iceland | 5 | 7 | 33288 |
| India | 421 | 6 | 564 |
| Indonesia | 328 | 1 | 1138 |
| Iran, Islamic Re | 78 | 1 | 2932 |
| Iraq | 86 | 2 | 4045 |
| Ireland | 9 | 1 | 28154 |
| Israel | 9 | 5 | 17781 |
| Italy | 6 | 7 | 23618 |
| Jamaica | 87 | 3 | 2976 |
| Japan | 11 | 7 | 32169 |
| Jordan | 88 | 3 | 2190 |
| Kazakhstan | 65 | 7 | 3400 |
| Kenya | 708 | 3 | 495 |
| Kiribati | 184 | 1 | 830 |
| Korea, Dem. Rep. | 100 | 7 | Data Missing |
| Korea, Rep. | 18 | 7 | 12118 |
| Kuwait | 7 | 4 | 22495 |
| Kyrgyz Republic | 83 | 7 | 524 |
| Lebanon | 49 | 1 | 4704 |
| Lesotho | 645 | 1 | 509 |
| Liberia | 1320 | 5 | 221 |
| Libya | 25 | 1 | 6909 |
| Lithuania | 21 | 7 | 6756 |
| Luxembourg | 12 | 6 | 55896 |
| Madagascar | 615 | 1 | 286 |
| Malawi | 872 | 1 | 205 |
| Malaysia | 66 | 3 | 4402 |
| Maldives | 374 | 2 | 2586 |
| Mali | 879 | 1 | 344 |
| Malta | 14 | 0 | 10949 |
| Mauritania | 815 | 1 | 658 |
| Mauritius | 64 | 1 | 4039 |
| Mexico | 75 | 4 | 5531 |
| Micronesia, Fed. | 158 | 1 | 2053 |
| Mongolia | 154 | 7 | 1168 |
| Morocco | 255 | 3 | 1551 |
| Mozambique | 1052 | 4 | 289 |
| Myanmar | 354 | 1 | 1107 |
| Nepal | 659 | 2 | 273 |
| Netherlands | 12 | 7 | 29922 |
| New Zealand | 15 | 5 | 18965 |
| Nicaragua | 190 | 1 | 932 |
| Niger | 812 | 1 | 250 |
| Nigeria | 1163 | 3 | 613 |
| Norway | 7 | 7 | 44890 |
| Oman | 25 | 1 | 9157 |
| Pakistan | 342 | 3 | 581 |
| Panama | 93 | 3 | 4032 |
| Papua New Guinea | 371 | 3 | 933 |
| Peru | 182 | 3 | 2277 |
| Philippines | 137 | 1 | 1107 |
| Poland | 11 | 6 | 6024 |
| Portugal | 15 | 5 | 12679 |
| Qatar | 24 | 4 | 32185 |
| Romania | 84 | 7 | 3231 |
| Russian Federati | 58 | 7 | 4607 |
| Rwanda | 1008 | 3 | 311 |
| Samoa | 115 | 3 | 1706 |
| Sao Tome and Pri | 252 | 4 | 924 |
| Senegal | 499 | 1 | 686 |
| Sierra Leone | 2412 | 3 | 276 |
| Singapore | 14 | 7 | 23574 |
| Slovenia | 12 | 7 | 15857 |
| Solomon Islands | 253 | 1 | 1118 |
| Somalia | 1091 | 1 | 521 |
| South Africa | 105 | 6 | 4044 |
| Spain | 6 | 5 | 17852 |
| Sri Lanka | 61 | 1 | 1019 |
| St. Vincent and the Grenadines, | 64 | 5 | 3617 |
| Sudan | 587 | 2 | 627 |
| Suriname | 185 | 1 | 3000 |
| Swaziland | 592 | 1 | 1711 |
| Sweden | 6 | 7 | 33576 |
| Switzerland | 7 | 4 | 45456 |
| Syrian Arab Repu | 92 | 1 | 1161 |
| Tajikistan | 87 | 7 | 365 |
| Tanzania | 847 | 3 | 342 |
| Thailand | 32 | 4 | 2472 |
| Togo | 510 | 5 | 366 |
| Tonga | 100 | 1 | 2008 |
| Tunisia | 100 | 7 | 2376 |
| Turkey | 77 | 7 | 4433 |
| Turkmenistan | 71 | 7 | 1602 |
| Uganda | 608 | 3 | 314 |
| Ukraine | 40 | 7 | 1557 |
| United Kingdom | 11 | 5 | 26415 |
| United States | 12 | 7 | 33463 |
| Uruguay | 33 | 4 | 5781 |
| Uzbekistan | 46 | 7 | 719 |
| Vanuatu | 168 | 3 | 1584 |
| Venezuela, RB | 94 | 1 | 4364 |
| Vietnam | 101 | 7 | 534 |
| Yemen, Rep. | 488 | 1 | 656 |
| Zambia | 497 | 5 | 582 |
| Zimbabwe | 516 | 4 | 622 |
