# Supplementary material for: Abortion laws reform may reduce maternal mortality: an ecological study in 162 countries
Source: BMC Womens Health. 2019 Jan 5;19:1. doi: 10.1186/s12905-018-0705-y (PMC6321671; doi:10.1186/s12905-018-0705-y)
Supplement: Supplementary file 2 — Glossary and Definitions. Glossary and Definitions for the Uncertainty Interval, maternal mortality ratio (MMR), Maternal Death, and Live Birth. (DOCX 33 kb) [file 12905_2018_705_MOESM2_ESM.docx]

**(Glossary/Definitions)**

**Uncertainty Interval (UI)**

The uncertainty intervals (UI) computed for all the estimates refer to the 80%uncertainty intervals (10th and 90th percentiles of the posterior distributions). This was chosen as opposed to the more standard 95% intervals because of the substantial uncertainty inherent in maternal mortality outcomes.

**Maternal mortality ratio (MMR)**

Maternal mortality ratio is defined as maternal deaths per 100,000 live births.

**Maternal Death**

Maternal death is the death of a woman which pregnant or within 42 days of termination of pregnancy, irrespective of the duration and site of the pregnancy, from any cause related to or aggravated by the pregnancy or its management but not from accidental or incidental causes.

**Live birth**

Live birth is the complete expulsion or extraction from its mother of a product of conception, irrespective of the duration of the pregnancy, which, after such separation, breathes or shows any other evidence of life- e.g. beating of the heart, pulsation of the umbilical cord has been cut or the placenta is attached. Each product of such a birth is considered live born.
