# Supplementary material for: Abortion laws reform may reduce maternal mortality: an ecological study in 162 countries
Source: BMC Womens Health. 2019 Jan 5;19:1. doi: 10.1186/s12905-018-0705-y (PMC6321671; doi:10.1186/s12905-018-0705-y)
Supplement: Supplementary file 3 — Maternal Mortality Ratios (Mean) and the flexibility score of abortion laws (Mean score) in the sample countries, 1985–2013. Description of Data: The table summarizes the average score for abortion laws and average maternal mortality ratios for each sample country from 1985 to 2013. (DOCX 37 kb) [file 12905_2018_705_MOESM3_ESM.docx]

**Table summarizing specific reasons for legal abortion for each flexibility score in sample countries**

| Score | Possible Combination | Percentage of possible combination |
| --- | --- | --- |
| 0 | No reason is allowed for legal abortion |  |
| 1 | Life Threatening | 100% |
| 2 | Life Threatening + Rape | 47% |
|  | Life Threatening + Physical Health | 36% |
|  | Life Threatening + Foetal Impairment | 17% |
| 3 | Life threat+ Physical health+ Mental health | 95% |
|  | Life threat+ Physical health + Rape | 3% |
|  | Life threat + Rape + Fetal impairment | 1% |
|  | Life threat+ Physical health + Fetal impairment | 1% |
| 4 | Life threat+ physical+ mental+ rape | 36% |
|  | Life threat+ physical + rape + fetal impairment | 26% |
|  | Life threat+ physical+ mental+ fetal impairment | 21% |
|  | Life threat+ physical+ mental+ economic | 8% |
|  | Life threat+ physical+ mental+ request | 7% |
|  | Life threat+ physical+ economic + rape | 1% |
| 5 | Life threat+ physical+ mental + rape+ fetal impairment | 80% |
|  | Life threat+ physical + mental + fetal impairment + economic | 20% |
| 6 | Life threat+ physical+ mental + rape+ fetal impairment +economic | 100% |
| 7 | Allowed for all 7 reasons |  |
