# Supplementary material for: Abortion laws reform may reduce maternal mortality: an ecological study in 162 countries
Source: BMC Womens Health. 2019 Jan 5;19:1. doi: 10.1186/s12905-018-0705-y (PMC6321671; doi:10.1186/s12905-018-0705-y)
Supplement: Supplementary file 4 — List of Latin-American and Caribbean Countries excluded for Sensitivity Analysis. The list provides the name of 24 Latin-America and Caribbean countries which has been excluded to conduct sensitivity analysis. (DOCX 35 kb) [file 12905_2018_705_MOESM4_ESM.docx]

**List of Latin-American and Caribbean Countries excluded for Sensitivity Analysis**

1. Argentina
2. Belize
3. Brazil
4. Chile
5. Colombia
6. Costa Rica
7. Cuba
8. Dominican Republic
9. Ecuador
10. El Salvador
11. Grenada
12. Guatemala
13. Guyana
14. Haiti
15. Honduras
16. Jamaica
17. Mexico
18. Nicaragua
19. Panama
20. Peru
21. Saint Vincent and the Grenadines
22. Suriname
23. Uruguay
24. Venezuela, RB
